# Tooth Grinding in Older Adults: A Retrospective Cross‐Sectional Study

**DOI:** 10.1111/scd.70092

**Published:** 2025-09-01

**Authors:** Leonardo Marchini, Jhanvi Desai, Fang Qian

**Affiliations:** ^1^ Department of Preventive and Community Dentistry The University of Iowa College of Dentistry and Dental Clinics Iowa City Iowa USA; ^2^ Division of Biostatistics and Computational Biology The University of Iowa College of Dentistry and Dental Clinics Iowa City Iowa USA

**Keywords:** aged, bruxism, older adults, self‐reported tooth grinding, sleep bruxism, tooth wear

## Abstract

**Objectives:**

This study aims to determine the prevalence of self‐reported tooth grinding among older adult patients at a dental school and to examine the associations between tooth grinding and various demographic and clinical factors.

**Methods:**

Data were retrieved from the electronic patient database at the University of Iowa College of Dentistry. Descriptive statistics and bivariate analyses were used to explore associations between tooth grinding and various demographic and clinical factors. Multivariable logistic regression was used to identify factors that were significantly associated with tooth grinding.

**Results:**

Out of 12,550 adults aged 65 years or older, 1598 (12.7%) who responded to the tooth grinding question were included in the analysis, with 853 (53.4%) reporting tooth grinding. Multivariable logistic regression analysis revealed that gender and drug addiction were significantly associated with tooth grinding, with males and those with a history of drug addiction having higher odds.

**Conclusions:**

In this large sample of older adults, self‐reported tooth grinding had a high prevalence, with males and those with a history of drug addiction having significantly higher odds of experiencing it.

## 1 Introduction

Bruxism has been defined in various ways by different authors [1, 2, 3], with the latest definition encompassing a range of jaw‐muscle activities during sleep or wakefulness. These activities can manifest as motor behaviors with or without teeth contact, such as clenching, grinding, bracing, or thrusting of the mandible. While some bruxism may be physiological, excessive bruxism could indicate an underlying condition or disorder and may have clinical consequences [4]. The precise etiologic mechanisms of bruxism remain unknown; however, numerous factors have been associated with its occurrence, including stress, neurotransmitter imbalances, parasomnias, mental health disorders, poor sleep hygiene, and a variety of medications [5, 6, 7, 8].

The diagnosis of bruxism has long been a subject of debate, lacking consensus. Recently, a standardized diagnostic tool was introduced to address this issue [9]. This comprehensive instrument is designed to evaluate bruxism status, risk factors, comorbid conditions, and consequences through multidimensional assessments, including self‐reports, clinical examinations, and technological evaluations. It consists of two axes covering 14 domains and 66 items, providing a thorough framework for both clinical and research applications [9]. However, this tool has not yet been widely implemented in clinical practice. Most clinical diagnoses of bruxism are based on observations of its consequences, such as excessive tooth wear, masticatory muscle hypertrophy, tooth/restoration fractures, temporomandibular disorder (TMD) signs and symptoms, and self‐reported teeth grinding or clenching [10, 11, 12].

Due in part to variations in diagnostic criteria, the reported prevalence of bruxism varies considerably across populations and age groups [13]. Estimates range from 3.5% to 40% among children [14], 8% to 31% among adults [15], and up to 25% in a sample of older adults in Germany [16]. The latter study also noted that the literature on bruxism prevalence in older adults remains relatively sparse. While symptoms of TMDs appear to be less common among older adults [16, 17, 18], individuals in this age group are more frequently affected by degenerative temporomandibular joint conditions, such as subchondral cysts and osteophytes [19], which may account for the increased prevalence of temporomandibular joint sounds reported in this population [17, 18].

Although symptomatic TMDs affect only a small proportion of the older adult population [16, 17, 18], bruxism remains a significant contributor to oral health problems in this age group [20]. Older adults are among the primary users of prosthodontic devices, and bruxism is a well‐recognized risk factor for mechanical and technical complications in prosthodontic rehabilitation. When prosthetic treatment is indicated in a patient with bruxism, it is generally recommended that steps be taken to mitigate the effects of potentially excessive occlusal loading on all components essential to the structural integrity of the prosthesis [20]. Given the importance of accurate bruxism assessment in older adults—and the central role that self‐reported tooth grinding plays in current diagnostic approaches [11, 21]—this study aims to determine the prevalence of self‐reported tooth grinding among older adult patients at a dental school clinic. Additionally, it seeks to examine the associations between self‐reported tooth grinding and a range of demographic and clinical variables.

## 2 Materials and Methods

This project was reviewed by the University of Iowa Institutional Review Board (IRB) and, because all data used in the analysis were de‐identified, it was classified as nonhuman subjects research under protocol #202503055. Out of the 12,550 active patients in the electronic health records (EHR) who were 65 years of age or older at the time their most recent health history update, 1598 (12.7%) who responded to the tooth grinding question were included in the analysis.

Descriptive statistics were utilized to summarize the demographic and clinical profiles of the patients. Categorical variables were expressed as frequencies and percentages, while continuous variables were characterized using means, standard deviations, medians, and interquartile ranges (IQRs). Bivariate analyses were conducted to explore the relationships between tooth grinding status (yes vs. no) and selected demographic and clinical factors. Depending on the type and distribution of the data, Pearson's Chi‐square test or Fisher's exact test was applied for categorical variables, and the nonparametric Wilcoxon rank‐sum test was used for continuous variables. The normality of continuous variables was assessed using the Shapiro–Wilk test to justify the application of nonparametric tests when necessary.

To identify significant predictors of tooth grinding, a multivariable logistic regression analysis was performed. Odds ratios (ORs) and 95% confidence intervals (95% CIs) were calculated for each predictor. In this study, four demographic variables were examined: gender, age, race/ethnicity, and type of insurance coverage. Eleven self‐reported clinical characteristics were also included based on previously reported associations with bruxism. [5, 6, 7, 8] These were: number of daily medications, BMI, tobacco use, alcohol addiction, drug addiction, breathing or lung problems, eating disorders, muscle disorders, neurological problems, diabetes, and mental health problems. Diabetes was excluded from the analysis due to a high percentage of missing data (over 70%). The model's fit was evaluated using the Hosmer–Lemeshow test to ensure it adequately represented the data. Multicollinearity among predictor variables was assessed using variance inflation factors (VIF) and tolerance values (TIF), and selected two‐way interactions were also examined.

All statistical tests were conducted at a significance level of 0.05. Analyses were performed using the SAS System, version 9.4 (SAS Institute Inc., Cary, NC, USA).

## 3 Results

Among the 1598 respondents, 853 (53.4%) reported experiencing tooth grinding, while 745 (46.6%) did not. Table 1 presents the demographic characteristics of older adults and their associations with tooth grinding. The majority of participants identified as White (96.3%), and 59.4% were female. The average age of participants was 71.8 years (±5.9), with a median age of 70 years (IQR: 67–75). Approximately 58.2% of participants had dental insurance. Bivariate analysis revealed a significant association between gender and tooth grinding (*p* < 0.001), with a higher prevalence among males (59.9%) compared to females (48.9%). Other demographic variables did not show significant associations with tooth grinding (*p* > 0.05).

**TABLE 1:** Demographic characteristics of adults aged 65 and older and their associations with tooth grinding.

		Presence of tooth grinding		
Characteristics	All participants (*N* = 1598) *n* (%)	Yes (*n* = 853) *n* (%)	No (*n *= 745) *n* (%)	*p* value
**Age (years)**				0.156
Mean ± SD (Range)	71.8 ± 5.9 (65–97)	71.7 ± 6.0 (65–97)	72.0 ± 5.8 (65–92)	
Median (IQR)	70 (67–75)	70 (67–75)	71 (67–75)	
**Age Group**				0.288
65–69 years	696 (43.5)	382 (54.9)	314 (45.1)	
70–79 years	720 (45.1)	383 (53.2)	337 (46.8)	
80–97 years	182 (11.4)	88 (48.4)	94 (51.6)	
**Gender**				**<0.001** ^a^
Female	949 (59.4)	464 (48.9)	485 (51.1)	
Male	649 (40.6)	389 (59.9)	260 (40.1)	
**Race‐Ethnicity**				0.260
White	1,152 (96.3)	623 (54.1)	529 (45.9)	
Non‐White	44 (3.7)	20 (45.5)	24 (54.5)	
**Types of insurance**				0.330
Self‐pay	668 (41.8)	347 (51.9)	321 (48.1)	
Non‐self‐pay (AG + DWP + INS + XIX)	930 (58.2)	506 (54.4)	424 (45.6)	

*Note*: Due to missing data, not all variables add up to the total sample size of 1598.

^a^ Statistically significant difference was observed between the two groups, as determined by the chi‐square test (*p* < 0.05).

Table 2 presents the clinical characteristics of participants and their associations with tooth grinding. Among the participants, 38.2% were classified as obese. The average number of daily medications was 9.0 (±7.3), with a median of 8 (IQR: 4–12). Tobacco use was reported by 45.6% of participants, while 48.7% and 10.2% reported histories of alcohol and drug addiction, respectively. Mental health problems were present in 34.6% of participants, and 32.9% had neurological conditions. Additionally, 44.2% reported breathing or lung problems, 2.0% had eating disorders, 61.6% had diabetes, and 43.6% experienced muscle disorders. The bivariate analysis indicated that none of the clinical characteristics were significantly associated with tooth grinding (*p* > 0.05 for all variables).

**TABLE 2:** Clinical characteristics of adults aged 65 and older and their associations with tooth grinding.

		Presence of tooth grinding		
Characteristics	All participants (*N* = 1598) *n* (%)	Yes (*n* = 853) *n* (%)	No (*n* = 745) *n* (%)	*p* value^a^
**BMI** (kg/m^2^)				0.510
Mean ± SD	29.1 ± 6.6	29.3 ± 6.7	28.8 ± 6.5	
Median (IQR)	28.3 (24.6–32.3)	28.5 (24.9–32.3)	27.9 (24.4–31.9)	
**BMI Level** (kg/m^2^)				0.509
<30 (Non‐obesity)	802 (61.8)	424 (52.9)	378 (47.1)	
≥30 (Obesity)	495 (38.2)	271 (54.7)	224 (45.3)	
**Number of daily medications**				0.257
Mean ± SD (Range)	9.0 ± 7.3 (0–71)	9.2 ± 7.5 (0–64)	8.8 ± 7.2 (0–71)	
Median (IQR)	8 (4–12)	8 (4–13)	7 (4–12)	
**Number of daily medications**				0.096
0—3	361 (22.6)	193 (53.5)	168 (46.5)	
4—9	626 (39.2)	315 (50.3)	311 (49.7)	
10+	611 (38.2)	345 (56.5)	266 (43.5)	
**Tobacco use**				0.993
Yes	588 (45.6)	324 (55.1)	264 (44.9)	
No	702 (54.4)	387 (55.1)	315 (44.9)	
**Alcohol addiction**				0.821
Yes	629 (48.7)	346 (55.0)	283 (45.0)	
No	662 (51.3)	360 (54.4)	302 (45.6)	
**Drug addiction**				0.283
Yes	132 (10.2)	67 (50.8)	65 (49.2)	
No	1157 (89.8)	644 (55.7)	513 (44.3)	
**Breathing or lung problems**				0.276
Yes	569 (44.2)	322 (56.6)	247 (43.4)	
No	719 (55.8)	385 (53.6)	334 (46.4)	
**Eating disorders**				0.735
Yes	25 (2.0)	13 (52.0)	12 (48.0)	
No	1240 (98.0)	687 (55.4)	553 (44.6)	
**Muscle disorders**				0.752
Yes	562 (43.6)	307 (54.6)	255 (45.4)	
No	726 (56.4)	403 (55.5)	323 (44.5)	
**Neurological problems**				0.854
Yes	421 (32.9)	231 (54.9)	190 (45.1)	
No	859 (67.1)	476 (55.4)	383 (44.6)	
**Mental health problems**				0.999
Yes	444 (34.6)	246 (55.4)	198 (44.6)	
No	841 (65.4)	466 (55.4)	375 (44.6)	

*Note*: Due to missing data, not all variables add up to the total sample size of 1598. The variable highlighted in red indicates substantial missing values.

^a^ No statistically significant differences were observed between the two groups, determined by either the Chi‐square test or the Wilcoxon rank‐sum test (*p* > 0.05).

For the multivariable logistic regression analysis, the model included fourteen independent variables: gender, age, race/ethnicity, type of insurance coverage, number of daily medications, BMI, tobacco use, alcohol addiction, drug addiction, breathing or lung problems, eating disorders, muscle disorders, neurological problems, and mental health problems.

After adjusting for other covariates in the model, gender and drug addiction were found to be statistically significantly associated with the odds of tooth grinding among older adults aged 65 years or older (Table 3). Specifically, males had 2.01 times the odds of experiencing tooth grinding compared to females (OR = 2.01, 95% CI: 1.45–2.77, *p* < 0.001). Regarding drug addiction, interestingly, while the bivariate analysis showed no statistically significant difference in the prevalence of tooth grinding between participants with and without a history of drug addiction (p = 0.283), the multivariable logistic regression model revealed a significant association. Specifically, participants with a history of drug addiction had significantly lower odds of tooth grinding compared to those without a history of drug addiction (OR = 0.56, 95% CI: 0.32–0.98, *p* = 0.041). This discrepancy suggests that confounding factors may have influenced the unadjusted association, and the adjusted model may better isolate the independent relationship between drug addiction and tooth grinding in this older adult population. Model fit was assessed using the Hosmer–Lemeshow Goodness‐of‐Fit test, yielding a nonsignificant result (*χ* ^2^ = 5.65, df = 8, *p* = 0.686), suggesting that the model adequately fits the data. In addition, multicollinearity among fourteen independent variables were examined, and no multicollinearity was detected. An interaction between age and the number of daily medications was also explored, but no significant interaction on tooth grinding was found, so the final model was not included the interaction term.

**TABLE 3:** Logistic regression predicting odds of tooth grinding among older adults aged 65 years or older.

Variables	OR (95% CI)^a^	*p* value
**Age (per year)**	0.99 (0.99–1.02)	0.449
**Gender** (male vs. female)	2.01 (1.45–2.77)	**<0.001** ^b^
**Race‐Ethnicity** (Non‐White vs. White)	0.88 (0.40–1.95)	0.760
**Insurance** (Non‐self‐pay vs. self‐pay)	1.22 (0.91–1.65)	0.181
**BMI (kg/m^2^)** (≥30 obesity vs. <30 non‐obesity)	0.99 (0.72–1.35)	0.944
**Number of daily medications taken**	1.02 (0.99–1.04)	0.095
**Tobacco use** (yes vs. no)	0.97 (0.72–1.31)	0.841
**Alcohol addiction** (yes vs. no)	1.01 (0.75–1.35)	0.975
**Drug addiction** (yes vs. no)	0.56 (0.32–0.98)	**0.041** ^b^
**Breathing or lung problem** (yes vs. no)	1.21 (0.88–1.68)	0.245
**Eating disorder** (yes vs. no)	0.95 (0.31–2.88)	0.926
**Muscle disorder** (yes vs. no)	1.17 (0.85–1.61)	0.336
**Neurological problem** (yes vs. no)	0.89 (0.64–1.25)	0.506
**Mental health problem** (yes vs. no)	1.20 (0.85–1.70)	0.293

*Note*: The Hosmer–Lemeshow Goodness of Fit test statistic = 5.65 with df = 8, *p *= 0.686.

^a^ OR, odds ratio; CI, 95% Wald Confidence Limits.

^b^ Statistically significantly associated with the outcome (*p *< 0.05).

## 4 Discussion

The prevalence of self‐reported tooth grinding among older adults in this sample of dental school patients was notably high, with slightly more than half of the respondents affirming the tooth‐grinding question. However, it is important to note that the vast majority (87.3%) of the original sample of older adult patients did not respond to this question. Inaccuracies and missing information have been frequently documented issues in self‐reported health history data, underscoring the need for integrated EHR [22, 23].

Nevertheless, the tooth‐grinding prevalence reported here is approximately double the prevalence of bruxism reported among older German adults in a recent study [16], and much higher than that reported in both a Swedish [24] and an Italian [25] older cohort. It also exceeds the high end (31%) of the previously reported range of bruxism prevalence for adults described in a systematic review [15]. However, it is important to highlight that self‐reported tooth grinding is not synonymous with bruxism. Self‐reported tooth grinding can lead to a high rate of false positive bruxism diagnoses [11], so the prevalence results presented here should be interpreted with caution.

In our cohort, self‐reported tooth grinding was significantly more prevalent among men. Although research on bruxism in older adults is limited [16], a Swedish study found that 70‐year‐old women reported bruxism problems at twice the rate (21%) of 80‐year‐old men (10%) [24]. An Italian study similarly observed a slightly higher prevalence of bruxism among women over 60 (29%) compared to men (25%) in the same age group [25]. Conversely, a recent German study found no significant gender differences in bruxism prevalence among older adults [16]. It is important to highlight again that self‐reported tooth grinding is not equivalent to bruxism. Additionally, our sample's reliance on responses from only 12.7% of patients may introduce response bias, and women generally report health issues more frequently than men [26].

In our logistic regression model, a history of drug addiction was significantly associated with a prevalence of self‐reported tooth grinding, alongside sex. The association between drug addiction history and tooth grinding observed in our study warrants careful interpretation. Individuals with a history of drug addiction were found to have significantly lower odds of reporting tooth grinding compared to those without such a history. This result contrasts with some prior research [7] and common clinical expectations, where substance use—particularly stimulant abuse—is often linked with bruxism or other oral parafunctional habits due to its impact on the central nervous system. [7] One possible explanation for our unexpected finding could be underreporting or denial of symptoms among individuals with a history of drug addiction, which may stem from stigma or reduced health‐seeking behavior. Alternatively, it is also possible that individuals with a history of addiction may have reduced awareness of oral symptoms. Another contributing factor could be that these individuals receive closer medical or dental monitoring and may be using medications (e.g., methadone, antipsychotics) that reduce bruxism. Further studies using longitudinal designs and detailed substance use histories may help clarify the nature of this relationship and account for potential confounding factors. In addition, older males have a higher prevalence of substance use and drug addiction especially to alcohol, opioids and illicit drugs when compared to older women [27]. Surprisingly, there was no significant correlation with the number of medications, despite many prescribed drugs also being associated with bruxism [7]. It is worth noting that the number of medications factor approached significance (*p* = 0.095) but did not reach it. Further studies are warranted to explore this association in greater detail.

This study is subject to several important limitations that warrant consideration. Chief among them is the issue of generalizability, as the findings are based on data from a single academic institution and may not accurately represent the broader older adult population. The reliance on EHR also introduces potential concerns regarding data integrity, including missing entries, inconsistent documentation, and possible inaccuracies. Moreover, the potential for selection bias cannot be overlooked, as individuals with more complete records or who are more responsive to specific survey items may differ systematically from those not captured as thoroughly. It is also essential to emphasize that the study's cross‐sectional and correlational design precludes any conclusions about causal relationships. Nonetheless, the large sample size strengthens the empirical value of the findings by contributing much‐needed data on the prevalence of self‐reported tooth grinding in older adults—a population for which current evidence remains limited. Future studies should aim for broader, nationally representative samples and adopt longitudinal designs to better assess causal pathways. Additionally, clinicians should routinely screen older adults for bruxism. Examining tooth wear and initiating a conversation can help uncover unrecognized grinding habits in this patient population.

## 5 Conclusion

In this extensive cohort of older adults, the incidence of self‐reported tooth grinding was notably high. Men and individuals with a history of substance abuse were significantly more likely to report experiencing this condition.

## Conflicts of Interest

The authors report there are no competing interests to declare.

## Data Availability

The data that support the findings of this study are available on request from the corresponding author. The data are not publicly available due to privacy or ethical restrictions.
